# Biological and Molecular Effects of Small Molecule Kinase Inhibitors on Low-Passage Human Colorectal Cancer Cell Lines

**DOI:** 10.1155/2014/568693

**Published:** 2014-09-17

**Authors:** Falko Lange, Benjamin Franz, Claudia Maletzki, Michael Linnebacher, Maja Hühns, Robert Jaster

**Affiliations:** ^1^Department of Medicine II, Division of Gastroenterology, Rostock University Medical Center, Ernst-Heydemann-Straße 6, 18057 Rostock, Germany; ^2^Oscar Langendorff Institute of Physiology, Rostock University Medical Center, Gertrudenstraße 9, 18057 Rostock, Germany; ^3^Molecular Oncology and Immunotherapy, Department of General Surgery, Rostock University Medical Center, Schillingallee 35, 18057 Rostock, Germany; ^4^Institute of Pathology, Rostock University Medical Center, Strempelstraße 14, 18055 Rostock, Germany

## Abstract

Low-passage cancer cell lines are versatile tools to study tumor cell biology. Here, we have employed four such cell lines, established from primary tumors of colorectal cancer (CRC) patients, to evaluate effects of the small molecule kinase inhibitors (SMI) vemurafenib, trametinib, perifosine, and regorafenib in an *in vitro* setting. The mutant *BRAF* (V600E/V600K) inhibitor vemurafenib, but also the MEK1/2 inhibitor trametinib efficiently inhibited DNA synthesis, signaling through ERK1/2 and expression of genes downstream of ERK1/2 in *BRAF* mutant cells only. In case of the AKT inhibitor perifosine, three cell lines showed a high or intermediate responsiveness to the drug while one cell line was resistant. The multikinase inhibitor regorafenib inhibited proliferation of all CRC lines with similar efficiency and independent of the presence or absence of *KRAS, BRAF, PIK3CA*, and *TP53* mutations. Regorafenib action was associated with broad-range inhibitory effects at the level of gene expression but not with a general inhibition of AKT or MEK/ERK signaling. In vemurafenib-sensitive cells, the antiproliferative effect of vemurafenib was enhanced by the other SMI. Together, our results provide insights into the determinants of SMI efficiencies in CRC cells and encourage the further use of low-passage CRC cell lines as preclinical models.

## 1. Introduction

Colorectal carcinoma (CRC) represents the third most common cancer in both sexes and the third leading cause of cancer-related deaths in the United States [1]. Despite considerable achievements in recent years, the therapeutic options in the locally advanced and metastatic stages of the disease still remain quite limited. For this reason, high hopes are associated with the clinical introduction of novel therapeutics that act by targeting protumorigenic mediators and intracellular signaling pathways. While monoclonal antibodies to vascular endothelial growth factor (VEGF) (bevacizumab) and the extracellular domain of epidermal growth factor receptor (EGFR) (cetuximab, panitumumab) are already established in treatment of advanced CRC [2], the application of small molecule kinase inhibitors (SMI) is still largely restricted to clinical trials. An important exception is the multikinase inhibitor regorafenib that blocks various angiogenic (VEGF receptor 1-3, TIE2), stromal (platelet-derived growth factor receptor-beta, fibroblast growth factor receptor), and oncogenic kinases (KIT, RET, and RAF) [3]. Regorafenib increases the overall survival of patients with metastatic CRC [4] and has been approved by the United States Food and Drug Administration in 2012. Various other SMI, many of them with more restricted targets than regorafenib, are currently in different phases of clinical testing.

In the transduction of proliferative and antiapoptotic signals in CRC cells, the signaling cascades RAS/RAF/MEK/ERK (extracellular signal regulated kinase) and PTEN (phosphatase and tensin homolog)/PI3K (phosphatidylinositol 3-kinase)/AKT/mTOR play pivotal roles [5, 6]. Both signaling pathways are activated by numerous growth factor receptors and mediate intracellular signals by the consecutive activation of downstream proteins. Upon activation by GTP-bound RAS, the serine/threonine kinase RAF triggers downstream signaling by phosphorylating MEK1 and MEK2, which in turn phosphorylate and activate ERK1 and ERK2. Activated ERKs may translocate into the nucleus where they phosphorylate transcription factors with key functions in the induction of cell proliferation and suppression of apoptosis [7, 8]. In CRC, activating mutations of the oncogenes* KRAS* and* BRAF* are observed in 30–60% [9, 10] and 10–15% [11], respectively. Oncogenic* KRAS* mutations are associated with resistance to EGFR inhibitors such as cetuximab [12].

PI3Ks are a family of lipid kinases that phosphorylate the 30-OH group on phosphatidylinositol in the plasma membrane. Subsequently, the serine/threonine kinase AKT is recruited to the cell membrane where it becomes phosphorylated and activated. In various types of cancer, the PI3K/AKT signaling cascade is critically involved in mediating survival and tumor cell growth [13, 14]. Furthermore, the PI3K/AKT signaling pathway is frequently activated in malignant tumors, including CRC, by growth factor receptor tyrosine kinases, by activating gene mutations of* KRAS* or* phosphatidylinositol-4,5-bisphosphate 3-kinase*,* catalytic subunit alpha *(*PIK3CA*), or by loss of function of the phosphatase PTEN [15, 16].

Here we have analyzed and compared the biological and molecular effects of four molecular cancer therapeutics, the multikinase inhibitor regorafenib [3], the inhibitor of the V600E/V600K mutant form of BRAF vemurafenib [17], the selective MEK1/MEK2 inhibitor trametinib [18], and the AKT inhibitor perifosine [19], in CRC cells in order to elucidate determinants of their efficiency or inefficiency.

Other than regorafenib, vemurafenib, trametinib, and perifosine are not established in the treatment of CRC. While BRAF-inhibitors such as vemurafenib have produced impressive response rates of approximately 60–80% in patients with* BRAF*-mutant metastatic malignant melanoma [20], vemurafenib is apparently much less efficient in* BRAF*-mutant CRC [21, meeting abstract]. As a possible mechanism of vemurafenib resistance, EGFR-mediated reactivation of ERK signaling has been proposed [22], but it has also been suggested that resistance to BRAF inhibition can be overcome with PI3K inhibition or demethylating agents [23]. In case of trametinib, encouraging preclinical data have been published that suggest direct antitumor activities on CRC cell lines both* in vitro* and* in vivo* [24] as well as an enhancement of the efficacy of 5-fluorouracil [25]. The results of clinical trials, however, are still awaited. Finally, perifosine was found to double the time to progression in one phase II trial for metastatic colon cancer [26], but later on failed its phase III clinical trial [27, meeting abstract]. On the other hand, perifosine has also been shown to act as a sensitizer to the anticolorectal cancer effects of curcumin; an effect that warrants further investigation [28]. Together, these findings indicate that all three drugs display inhibitory effects on CRC cells in preclinical settings but also illustrate that their clinical efficiency is either still unknown or questionable. Therefore, the use of these substances in our studies was most of all motivated by their molecular specificity and not their (uncertain) clinical efficiency.

In our studies, we took advantage of a panel of recently established low-passage cell lines that were derived from primary tumors of surgical CRC patients [29, 30]. In contrast to cell lines of high passage [31, 32], low-passage cancer cell lines well reflect the biology of the original tumor, such as growth behavior, morphology, and mutational profile and are, therefore, in our experience, a versatile tool to evaluate drug efficiencies in a preclinical context. To reflect the three molecular classes of CRC, cell lines with chromosomal instability (CIN), microsatellite instability (MSI), and a CpG island methylator phenotype were included into the investigations. With respect to CRC-typical molecular alterations, the cell lines were characterized by an individual, only partially overlapping molecular profile that included oncogenic mutations of* KRAS*,* BRAF* (V600E), and* PIK3CA* as well as loss or inactivation of the tumor suppressor genes* APC* and* TP53*. The study, therefore, also aimed at a systematic evaluation of relationships between the biological efficiency of the investigated SMI, the mutational profiles of the CRC cells, and the activity of downstream signaling pathways and target genes.

The results show that the efficacy of vemurafenib and trametinib in CRC cells depends on the presence of mutant* BRAF* (V600E) and an efficient inhibition of MEK/ERK signaling, whereas regorafenib action was largely independent of the molecular status of the cells and perifosine showed a cell line-specific action profile.

## 2. Materials and Methods

### 2.1. Reagents

Unless stated otherwise, all reagents were obtained from Sigma-Aldrich (Deisenhofen, Germany).

### 2.2. Cell Line Establishment Protocol and Cell Culture

Primary CRC resection specimens were obtained from surgery, with informed written patient consent. All procedures were approved by the Ethics Committee of the University of Rostock (reference number II HV 43/2004) in accordance with generally accepted guidelines for the use of human material. Establishment of the cell line HROC24 has been described before [30]. The other cell lines were either directly established from fresh tumor material (HROC18 and HROC43) or following xenografting (HROC46) in immunodeficient NMRI-*Foxn1* ^*nu*^ mice. Cell line establishment protocol was adapted according to [30]. Briefly, single cell suspensions were seeded on collagen-coated plates in Dulbecco's MEM/Ham's F-12 (Biochrom, Berlin, Germany) supplemented with 10% fetal calf serum (FCS), 100 U/mL penicillin, and 100 *μ*g/mL streptomycin (complete culture medium; all reagents from PAA Laboratories, Pasching, Austria) at 37°C in a 5% CO_2_ humidified atmosphere. Continually growing cell cultures were regularly passaged. Cell lines used in this study did not exceed passage 50. Clinical, pathological, and molecular characteristics of the patients are summarized in Table 1. Noteworthy, all four cell lines were mutant for* APC* and wild-type for* PTEN*. One cell line (HROC24) expressed mutant* BRAF* (V600E), two cell lines (HROC43 and HROC46) oncogenic* KRAS*, and also two cell lines (HROC24 and HROC46) were mutant for* TP53*. HROC18 is the only cell line which harbors an E545K mutation of* PIK3CA* that increases the catalytic activity of the protein [33].

### 2.3. Quantification of DNA Synthesis

To analyze the effects of the SMI vemurafenib, perifosine, regorafenib, and trametinib (all from Selleckchem, Houston, TX, USA) on cell proliferation, DNA synthesis was measured using a 5-bromo-2′-deoxy-uridine (BrdU) incorporation assay kit (Roche Applied Science, Mannheim, Germany). Therefore, cells of the indicated CRC lines were plated in 96-well half-area microplates at equal seeding densities and allowed to adhere overnight in complete culture medium. The next day, the cells were serum-starved for 16 h before the FCS-free medium was substituted by complete culture medium supplemented with kinase inhibitors as indicated. After an incubation period of 24 h, BrdU labeling was initiated by adding labeling solution at a final concentration of 10 *μ*M. Another 8 h later, labeling was stopped and BrdU uptake was measured according to the manufacturer's instructions. IC_50_ values were determined by interpolation from the dose response curves.

### 2.4. Detection of Dead Cells and Analysis of Cellular DNA Content by Flow Cytometry

HROC24 cells growing in 12-well plates in complete culture medium were exposed to SMI and combinations thereof for 48 h as indicated. Afterwards, the cells were harvested by trypsinization, resuspended in buffer for flow cytometry (PBS pH 7.4; 0.5% bovine serum albumin; 0.1% sodium azide) and kept on ice until measurement. Subsequently, the samples were labeled with propidium iodide (PI; 10 *μ*g/mL). PI-positive (dead) cells were quantified using a FACSCalibur cytometer (BD Biosciences, Heidelberg, Germany).

In addition, cell death was verified by trypan blue staining of trypsinized cells as an independent method.

For the detection of the cellular DNA content, trypsinized HROC24 cells were pelleted by centrifugation, washed twice with PBS (pH 7.4), and resuspended in ice-cold 70% ethanol for at least 12 h at 4°C. After additional washing steps, the cells were incubated for 20 min in 400 *μ*L PBS supplemented with 0.1 mg/mL RNase A (Roche Applied Science) at 37°C. Subsequently, 50 *μ*g/mL PI was added and the samples were subjected to cytofluorometric analysis. 10.000 events were measured for each sample and the data stored in list mode for further analysis. The cell cycle distribution was calculated using the software tool* Cyflogic* (CyFlo Ltd, Finland). Cells of the Sub-G1 peak were considered apoptotic.

### 2.5. Immunoblotting

Cells of the indicated CRC lines were grown in 24-well plates in complete culture medium until reaching subconfluency before they were treated with SMI for 6 h. Afterwards, protein extracts were prepared and subjected to immunoblot analysis as published before [34], using polyvinylidene fluoride membrane for protein transfer. The following primary antibodies (all from New England BioLabs, Frankfurt, Germany, unless specified otherwise) were employed: anti-GAPDH (#2118), anti-phospho-AKT (P-AKT; #4060), anti-phospho-MEK (P-MEK1/2; #9154), anti-phospho-ERK1/2 (P-ERK1/2) (#4370), anti-AKT protein (#4691), anti-MEK1/2 (#8727), and anti-ERK1/2 (#06-182, Millipore, Billerica, MA, United States). The blots were developed using LI-COR reagents for an Odyssey Infrared Imaging System as previously described [35]. The signal intensities of the investigated proteins were quantified by means of the Odyssey software and raw data processed as described in the corresponding figure legend.

### 2.6. Quantitative Reverse Transcriptase-PCR Using Real-Time TaqMan Technology

HROC24 cells growing in 12-well plates were treated with SMI for 6 h as indicated. Afterwards, total RNA was isolated with TriFast reagent (PEQLAB Biotechnologie, Erlangen, Germany) according to the manufacturer's instructions. All further steps were performed with reagents from Life Technologies (Darmstadt, Germany). First, any traces of genomic DNA were removed employing the DNA-free kit. Next, 1 *μ*g of RNA was reverse transcribed into cDNA by means of TaqMan Reverse Transcription Reagents and random hexamer priming. Relative quantification of target cDNA levels by real-time PCR was performed in an ABI Prism 7000 sequence detection system (Life Technologies). Therefore, TaqMan Universal PCR Master Mix and human gene-specific Assay-on-Demand kits with fluorescently labeled MGB probes were used. The following assays were employed: Hs99999140_m1 (*FOS*), Hs00355782_m1 (*CDKN1A*;* p21*), Hs00244839_m1 (*DUSP5*), Hs00180269_m1 (*BAX*), Hs00181225_m1 (*FAS ligand*;* FASLG*;* CD95L*), Hs01034249_m1 (*TP53*), Hs00765553_m1 (*cyclin D1*;* CCND1*), Hs00608023_m1 (*BCL2*), and Hs99999905_m1 (*GAPDH*; house-keeping gene control). PCR conditions were 95°C for 10 min, followed by 40 cycles of 15 s at 95°C/1 min at 60°C. The relative expression of each mRNA (*n* = 4–8 independent samples per experimental condition) compared with* GAPDH* was calculated according to the equation ΔCt = Ct_target_ − Ct_GAPDH_. The relative amount of target mRNA in control cells and cells treated with kinase inhibitors as indicated was expressed as 2^−(ΔCt)^.

### 2.7. Statistical Analysis

Values are expressed as mean ± standard error of the mean (SEM) for the indicated number of separate cultures per experimental protocol. Statistical significance was checked using the Mann-Whitney *U* test. *P* < 0.05 (Bonferroni-adjusted as indicated in the figure legends) was considered to be statistically significant.

## 3. Results

### 3.1. Antiproliferative Effects of SMI on CRC Low-Passage Cell Lines

**Figure 1:**
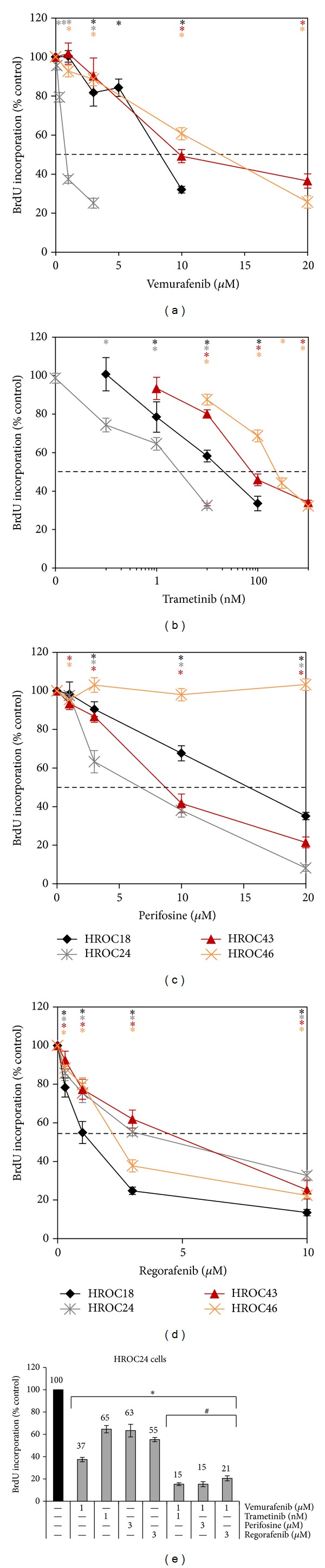
Effects of SMI on the BrdU incorporation of CRC cell lines. HROC18, HROC24, HROC43, and HROC46 cells growing in 96-well half-area microplates were treated with (a) vemurafenib, (b) trametinib (please note the logarithmic scale *x*-axis), (c) perifosine, and (d) regorafenib as indicated for 24 h, before DNA synthesis was assessed with the BrdU incorporation assay. In (e), HROC24 cells were incubated with combinations of SMI as indicated. One hundred percent BrdU incorporation corresponds to cells cultured without SMI. Data are presented as mean ± SEM (*n* ≥ 12 separate cultures); *P < 0.004 versus control cultures with Bonferroni-adjusted *α* = 0.0125; ^#^ *P* < 0.001 versus cultures treated with either of the two combined substances alone with Bonferroni-adjusted *α* = 0.0038.

In initial studies, the four low-passage CRC cell lines were exposed to different doses of vemurafenib, trametinib, perifosine, and regorafenib, respectively, and cell proliferation was assessed by measuring the incorporation of BrdU into newly synthesized DNA (Figure 1).

As expected, the inhibitor of mutant BRAF, vemurafenib, efficiently inhibited DNA synthesis of HROC24 cells, the only cell line in this study that harbors the V600E* BRAF* oncogene (IC_50_ = 0.8 *μ*M; Figure 1(a), Table 2). In contrast, for the other three cell lines IC_50_ values from 8.3–12.7 *μ*M were determined. HROC24 was also the only cell line that was sensitive to low nanomolar (and even subnanomolar) concentrations of the MEK1/2 inhibitor trametinib (IC_50_ = 5.1 nM; Figure 1(b)). Of the other three CRC lines, HROC18 showed the highest sensitivity to trametinib (IC_50_ = 39.9 nM), HROC43 showed an intermediate responsiveness (IC_50_ = 89.2 nM), and HROC46 showed the poorest response (IC_50_ = 252 nM).

The AKT inhibitor perifosine inhibited proliferation of the CRC lines HROC24 and HROC43 with similar efficiency (IC_50_ = 6.7 *μ*M and 8.7 *μ*M, resp.), while HROC18 cells were less sensitive (IC_50_ = 15.4 *μ*M) and HROC46 cells were resistant to the drug (Figure 1(c); small nonsystematic decrease at 1 *μ*M only).

The response pattern to the multikinase inhibitor regorafenib differed from the one to all other drugs in that (i) similar IC_50_ values were determined for all four cell lines (ranging from 1.3 to 5.3 *μ*M; Figure 1(d)) and (ii) HROC18 but not HROC24 was the most sensitive cell line.

In subsequent experiments, effects of SMI combinations were investigated employing HROC24 cells. The results show an additive action of vemurafenib when combined with any of the other three drugs (Figure 1(e)). In contrast, the simultaneous application of perifosine and trametinib did not result in additive effects of the drugs in any of the four CRC cell lines (data not shown).

### 3.2. Induction of Cell Death by SMI

**Figure 2:**
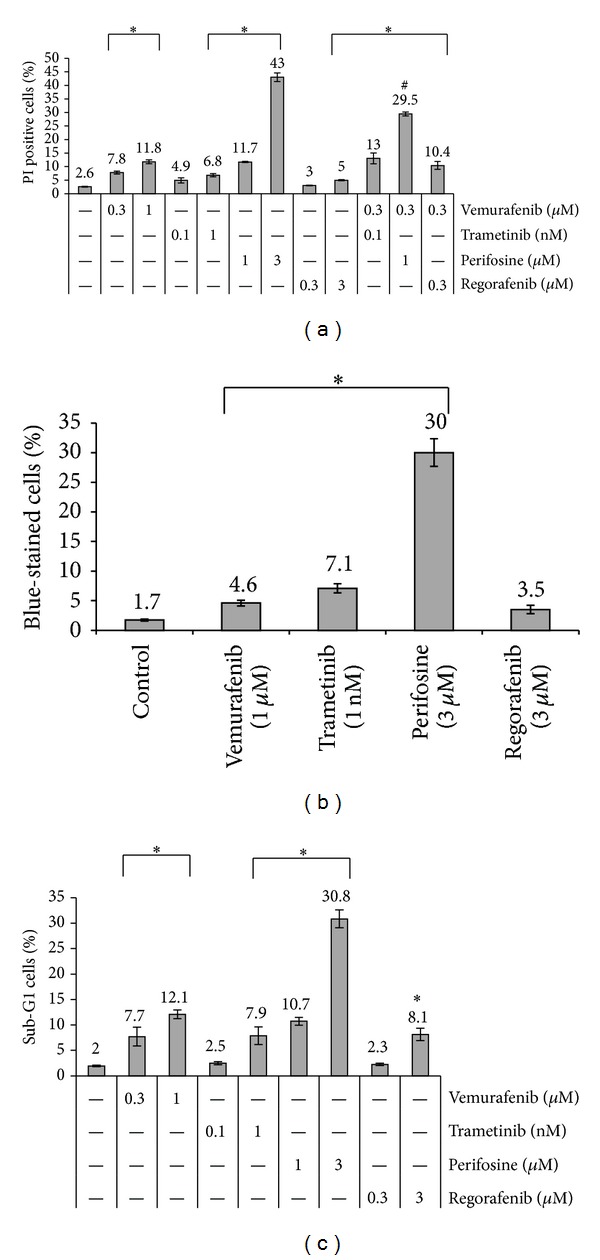
SMI-dependent death of HROC24 cells. HROC24 cells growing in 12-well plates were treated with SMI and combinations thereof for 48 h as indicated. Afterwards, they were subjected to (a) cytofluorometric quantification of dead (PI-positive) cells, (b) determination of cell death by trypan blue staining, and (c) detection of apoptotic (Sub-G1 peak) cells. The portion of dead/apoptotic cells is expressed as percent of the total cell count. Data from *n* ≥ 6 separate cultures were used to calculate mean values ± SEM. (a): *P < 0.001 versus control cells cultured without SMI and ^#^ *P* < 0.002 versus cells cultured with single SMI with Bonferroni-adjusted *α* = 0.00294; (b): *P < 0.008 versus control cultures with Bonferroni-adjusted *α* = 0.0125; (c): *P < 0.001 versus control cultures with Bonferroni-adjusted *α* = 0.00625.

For each SMI, the effects of two concentrations (selected based on the BrdU incorporation data) on cell death were determined by FACS analysis. As shown in Figure 2(a) for HROC24 cells, both concentrations of vemurafenib and perifosine as well as the higher concentrations of regorafenib and trametinib significantly increased the portion of PI-positive (dead) cells. For the higher SMI concentrations, these findings were largely confirmed by the results of trypan blue staining, except for that the effect of regorafenib was insignificant (Figure 2(b)). However, only perifosine (at 3 *μ*M) but none of the other inhibitors caused the death of more than 12% of the cells (PI-staining data; Figure 2(a)). Furthermore, when vemurafenib was applied together with low doses of the other drugs, only its combination with perifosine significantly increased its cytostatic effects (Figure 2(a)).

To further analyze the mechanisms of cell death, apoptotic cells were quantified employing the Sub-G1 peak method. As shown in Figure 2(c), for each inhibitor, a portion of apoptotic cells was determined that largely overlapped with the portion of PI-positive cells (Figure 2(a)). Together, these data suggest apoptosis as the main cause of CRC cell death under the given experimental conditions.

### 3.3. Effects of SMI on RAS/RAF/MEK/ERK and PI3K/AKT Signaling in CRC Cells

**Figure 3:**
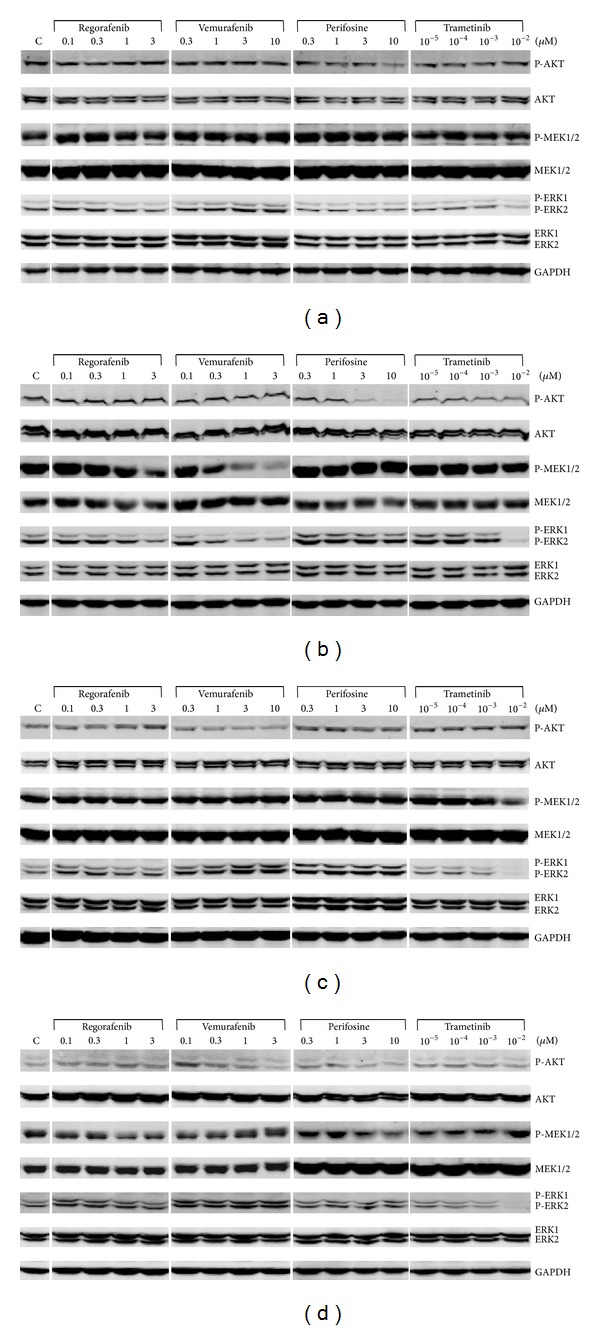
Effects of vemurafenib, trametinib, perifosine, and regorafenib on the phosphorylation of AKT, MEK1/2, and ERK1/2 in CRC cell lines. (a) HROC18, (b) HROC24, (c) HROC43, and (d) HROC46 cells were grown in 24-well plates to subconfluency before culture medium was supplemented with vemurafenib, trametinib, perifosine, and regorafenib at the indicated concentrations. Control cultures (C) were treated with solvent only. After an incubation period of 6 h, protein extracts from equal amounts of cells were subjected to immunoblot analysis. P-AKT, P-MEK1/2, P-ERK1/2, the respective total proteins, and GAPDH (for loading control) were detected using fluorescein- (IRDye-) labeled secondary antibodies. For each cell line, one representative blot is shown. For mean values of independent experiments, please refer to Figure 4.

**Figure 4:**
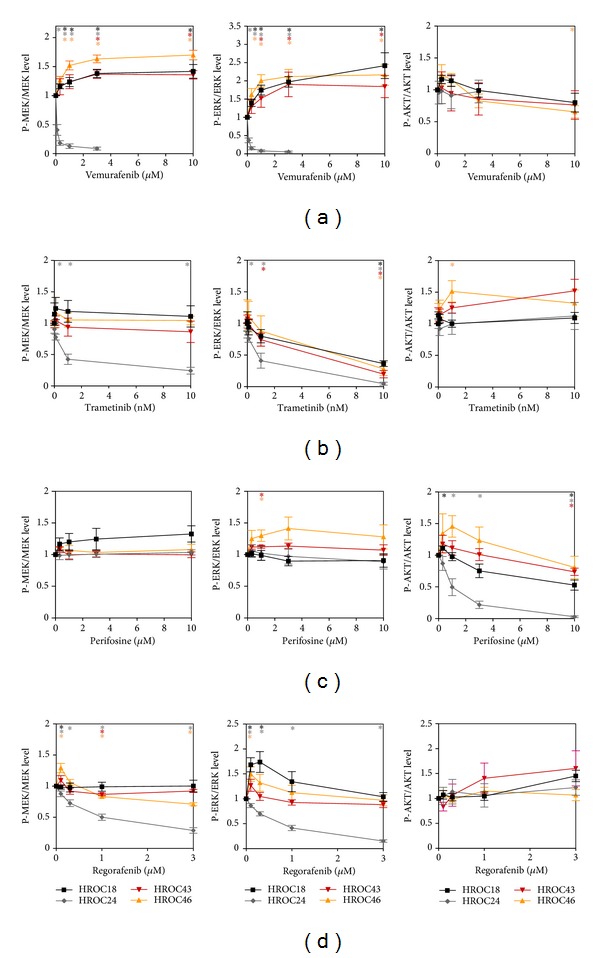
Quantitative analysis of the effects of vemurafenib, trametinib, perifosine, and regorafenib on signal transduction in CRC cell lines. The effects of (a) vemurafenib, (b) trametinib, (c) perifosine, and (d) regorafenib on fluorescence signal intensities of phosphoproteins (P-AKT, P-MEK1/2, and P-ERK1/2, resp.) and corresponding total proteins in HROC18 (black), HROC24 (grey), HROC43 (red), and HROC46 (orange) cells were quantified. Subsequently, the ratios P-MEK/MEK protein (left panels), P-ERK/ERK protein (middle panels), and P-AKT/AKT protein (right panels) were determined. A ratio of 1 corresponds to control cells cultured without SMI. Data of 5 independent experiments were used to calculate mean values ± SEM; *P < 0.01 versus control cultures with Bonferroni-adjusted *α* = 0.0125.

We next studied how the four investigated SMI affected expression and phosphorylation of AKT, MEK1/2, and ERK1/2 in HROC18, HROC24, HROC43, and HROC46 cells. For each cell line, typical immunoblots are shown in Figures 3(a)–3(d), while the quantitative effects of the four SMI are presented in Figures 4(a)–4(d).

In agreement with its profile of biological activities, vemurafenib displayed consistent inhibitory effects exclusively on the* BRAF*-mutant HROC24 cells, where it efficiently blocked phosphorylation of MEK1/2 and ERK1/2 (Figure 4(a)) at concentrations that also significantly diminished cell growth. For the other three cell lines, even increases of P-MEK1/2 and P-ERK1/2 levels were observed. Similar to vemurafenib, trametinib inhibited MEK1/2 phosphorylation in HROC24 cells only (Figure 4(b)). However, since trametinib specifically inhibits MEK activity, not phosphorylation, the more meaningful findings refer to the phosphorylation of ERK1/2. Here, a dose-dependent inhibitory effect of the drug was observed in all four types of CRC cells, with HROC24, like in the biological assays, as the most sensitive cell line. Significant changes of P-AKT levels in response to vemurafenib and trametinib treatment were restricted to HROC46 cells, where vemurafenib at 10 *μ*M caused a decrease and trametinib at 1 nM caused an increase of the P-AKT/AKT ratio.

As expected, the AKT inhibitor perifosine did not reduce MEK1/2 and ERK1/2 phosphorylation in any of the four cell lines (Figure 4(c)); in HROC43 and HROC46 cells* increased* P-ERK levels at a perifosine concentration of 1 *μ*M were detected. In agreement with the biological data (Figure 1), perifosine inhibited phosphorylation of AKT in the susceptible cell line HROC24 but was inefficient in the resistant cell line HROC46. Like in the BrdU incorporation assay, HROC18 and HROC43 displayed an intermediate sensitivity.

Although regorafenib reduced DNA synthesis in all four CRC lines (Figure 1), it consistently diminished phosphorylation of MEK1/2 and ERK1/2 in HROC24 cells only (Figure 4(d)). For the other three cell lines, the occasional significant effects did not follow a systematic pattern. Phosphorylation of AKT was not inhibited in any of the cell lines tested.

### 3.4. SMI Target Genes in HROC24 Cells

**Figure 5:**
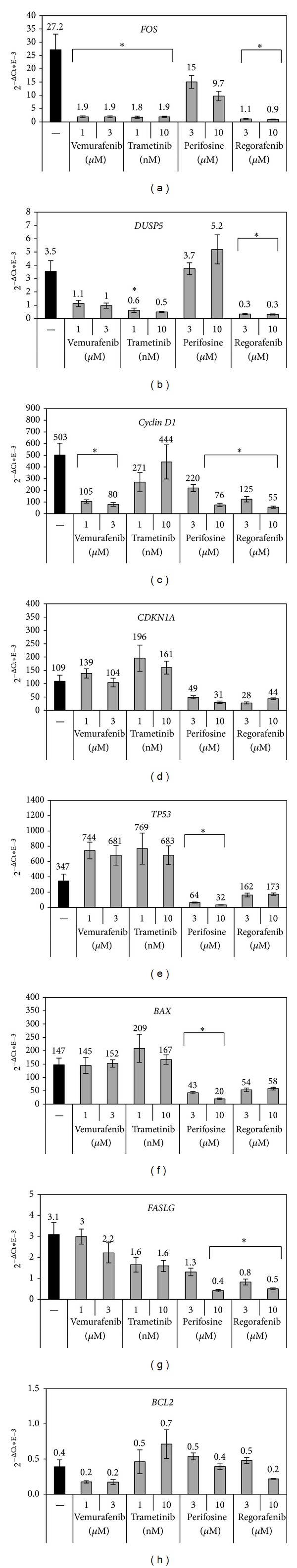
Gene expression profiles of SMI-treated HROC24 cells. Cultured HROC24 cells were exposed to SMI at the indicated concentrations for 6 hours. The mRNA expression of (a)* FOS*, (b)* DUSP5*, (c)* cyclin D1*, (d)* CDKN1A*, (e)* TP53*, (f)* BAX*, (g)* FASLG*, and (h)* BCL2* and the housekeeping gene* HPRT* was analyzed by real-time PCR and relative amounts of target mRNA were calculated as described in the “materials and methods” section. Data of *n* = 4–8 independent cultures were used to calculate mean values ± SEM. *P < 0.006 versus control cultures with Bonferroni-adjusted *α* = 0.00625.

Using the CRC line HROC24, we also studied molecular effects of the investigated SMI at the level of gene expression. Therefore, a panel was chosen that covered target genes of RAS/RAF/MEK/ERK signaling (*FOS*,* DUSP5*), stimulators (*cyclin D1*/*CCND1*), and inhibitors (*CDKN1A*,* TP53*) of cell cycle progression as well as proapoptotic (*BAX*,* FASLG*) and antiapoptotic (*BCL2*) effectors. The results (Figure 5) indicate that the four SMI can be divided into two groups with different action profiles.

Vemurafenib and trametinib strongly inhibited the expression of* FOS* (Figure 5(a)) but caused no statistically significant changes of the mRNA levels of* CDKN1A* (d),* TP53* (e),* BAX* (f),* FASLG* (g), and* BCL2* (h). Partially discordant results were observed for* DUSP5* (b) and* cyclin D1* (c), where either only trametinib (at 1 nM;* DUSP5*) or vemurafenib (*cyclin D1*) displayed significant inhibitory effects (although the other drug showed by trend a similar effect in both cases). The other two SMI, perifosine and regorafenib, changed the mRNA levels of a larger panel of genes. In case of regorafenib, statistically significant inhibitory effects on the expression of* FOS* (a),* DUSP5* (b),* cyclin D1* (c), and* FASLG* (g) as well as a trend to a reduced expression of several other genes were observed. Perifosine diminished the mRNA levels of* cyclin D1* (c),* TP53* (e),* FASLG* (g), and* BAX* (h).

## 4. Discussion

Low-passage human cancer cell lines are increasingly acknowledged as advantageous preclinical models for testing drug efficiencies and analyzing the molecular basis of drug sensitivity and resistance [36, 37]. Compared to long-term established high-passage cell lines, they more closely resemble their parental primary cancers regarding genotype and phenotypic features and, therefore, offer improved chances to address clinically relevant questions in the field of cancer medicine [30–32, 36, 37]. Here, we took advantage of four low-passage CRC cell lines with well-defined molecular phenotypes [29, 30] to evaluate the biological and molecular effects of selected SMI that interfere with signaling through two key mitogenic/antiapoptotic pathways in CRC cells, RAS/RAF/MEK/ERK, and PTEN/PI3K/AKT/mTOR. The studies were motivated by the fact that many drugs deemed active against a particular type of cancer are effective in a subset of patients only. To this end, however, predictive molecular biomarkers to stratify cancer patients for treatment are available in exceptional cases only (e.g., absence of oncogenic* KRAS* mutations as a prerequisite for treatment of CRC patients with anti-EGFR antibodies [12]).

Vemurafenib acts as specific inhibitor of the V600E/V600K mutant form of BRAF [17] and was therefore predicted to selectively target HROC24 cells, the only mutant* BRAF* cell line used in this study. Indeed, both biological and molecular data (Figures 1 and 4, resp.) pointed to a unique sensitivity of HROC24 cells to the drug. We considered these expected results as further support for our concept to identify links between the biological sensitivity of low-passage CRC lines and specific molecular alterations. Previous studies in commonly used high-passage* BRAF*-mutant lines have suggested that CRC cells are much less sensitive to vemurafenib than malignant melanoma cells due to an EGFR-mediated reactivation of ERK signaling [22]. Although our data are not contradictory to these findings, it is still interesting to note that vemurafenib almost completely blocked phosphorylation of MEK1/2 and ERK1/2 in HROC24 cells over at least 6 h at low micromolar concentrations.

In case of the specific MEK1/2 inhibitor trametinib [18], a graduated response of the four CRC cell lines was observed. Again, HROC24 cells were much more sensitive to the drug than the other three cell lines both at the levels of DNA synthesis (Figure 1) and signal transduction (Figure 4); a finding that is compatible with a strict dependency of HROC24 cells on a constitutive activation of the MEK/ERK signaling pathway by mutant BRAF. Unexpectedly, the only remaining wild-type* KRAS* cell line, HROC18, displayed the second-lowest IC_50_ value for trametinib in the BrdU incorporation assay, while the two CRC lines with oncogenic* KRAS* mutations, HROC43 and HROC46, were less sensitive. In HROC18, HROC43, and HROC46 cells, suppression of DNA synthesis did not correlate with the inhibition of ERK phosphorylation, which showed a similar dose dependency in all three cell lines. Finally, the* TP53* status was no predictor of the trametinib responsiveness. Together, our data suggest the presence of oncogenic* BRAF* as determinant of the efficiency of trametinib in CRC cells, an observation that is in line with similar findings in malignant melanoma [20]. At the level of gene expression, the effects of both vemurafenib and trametinib were largely consistent with the action profile of drugs that act by targeting the RAS/RAF/MEK/ERK signaling pathways. Thus, both drugs strongly inhibited the expression of the* FOS* gene, a key regulator of cell proliferation, differentiation, and survival that is transcriptionally regulated by the aforementioned signaling cascade [38].

For the AKT inhibitor perifosine [19], a comparison of the most sensitive cell line, HROC24, with largely resistant HROC46 cells revealed a correlation between the inhibition of DNA synthesis and reduction of AKT phosphorylation. On the other hand, the molecular basis of the complete biological resistance of HROC46 cells warrants further investigations, since* PTEN* mutations were not detected and at least by trend a decrease of P-AKT levels at a perifosine concentration of 10 *μ*M was observed. Noteworthy, HROC18, the only cell line in our investigation that carries the E545K mutation of* PIK3CA*, displayed an intermediate sensitivity to perifosine only.

In this study, perifosine was the only SMI that strongly affected cell survival by inducing apoptosis (shown for HROC24 cells; Figure 2). At the level of gene expression, however, the effects of perifosine were only in part in line with its proapoptotic efficiency (diminished levels of* cyclin D1*). Other effects of perifosine (inhibition of* TP53*,* BAX*, and* FASLG* expression) were unexpected and require follow-up studies for interpretation.

The multikinase inhibitor regorafenib [3] diminished the proliferation of all four CRC lines with similar efficiency (IC_50_ values in the low micromolar range). Given that cell lines of all three molecular classes of CRC and with a different mutation status of* KRAS*,* BRAF*,* PIK3CA*, and* TP53* were studied, these data suggest regorafenib efficiency as quite robust against specific molecular alterations. At the level of gene expression, a broad-range inhibitory effect of regorafenib was observed that fits its action profile as a multikinase inhibitor. Surprisingly, however, regorafenib inhibited phosphorylation of MEK1/2 and ERK1/2 exclusively in HROC24 cells and AKT phosphorylation even not at all. We therefore hypothesize that both signaling pathways are not essential for the inhibition of DNA synthesis and gene expression in low-passage HROC cells by regorafenib.

Interestingly, the combination of vemurafenib with any of the other three drugs resulted in additive inhibitory effects on the proliferation of HROC24 cells. Combination therapy with BRAF and MEK inhibition is currently in clinical development for the treatment of* BRAF* mutated malignant melanoma [39]. Based on our data, we suggest that further preclinical studies should address the question if CRC might be another suitable target for such a combination of drugs. We expand this conclusion to the simultaneous application of vemurafenib and regorafenib or a specific AKT inhibitor, since all these drug combinations showed similar potencies in our assays. As a next step, we are planning* in vivo* studies in mice with xenografted tumors to validate and expand the results of our* in vitro* investigations.

## 5. Conclusions

Together, the results of this study have provided novel insights into the molecular determinants of SMI efficiencies in CRC cells. Specifically, a MSI-positive cell line with mutant* BRAF*, HROC24, was most sensitive not only to vemurafenib but also to trametinib and perifosine treatment. The multikinase inhibitor regorafenib displayed growth-inhibitory effects that were largely independent of the mutational profile and the molecular class of the tumor. Combinations of regorafenib with specific SMI such as vemurafenib (in* BRAF*-mutant tumors), trametinib and perifosine warrant further evaluation. Low-passage cell lines, like the ones used in this study, are relevant preclinical models and therefore advantageous for the testing of novel targeted therapeutics.

## Figures and Tables

**Table 1:** Clinical and pathological characteristics of patients and HROC cell lines.

Tumor ID	Age/Gender	Tumor location	TNM-Stage	UICC stage	Tumor type	Molecular type	Molecular alterations					
							*APC *	*TP53 *	*KRAS *	*BRAF *	*PIK3CA *	*PTEN *
HROC18	65/f	caecum	G2T2N0M0 R0L0V0	I	Primary adenocarcinoma	spStd	mut	mut	wt	wt	mut	wt
HROC24	98/m	colon ascendens	G2T2N0M0 R0L0V1	I	Primary adenocarcinoma	spMSI-H	mut	wt	wt	mut	wt	wt
HROC43	72/m	colon ascendens	G3T3N2M0 R0L1V0	IIIb	Primary adenocarcinoma	CIMP-L	mut	mut	mut	wt	wt	wt
HROC46∗	66/m	colon ascendens	G3T3N0M1 R2L0V1	IV	Primary adenocarcinoma	spStd	mut	wt	mut	wt	wt	wt

f: female, m: male, spStd**: **sporadic standard, spMSI: sporadic microsatellite instable, CIMP-L: CpG island methylator phenotype, and L**: **low, H**: **high. ∗Xenograft-derived cell line.

**Table 2:** IC_50_ values of vemurafenib, perifosine, regorafenib, and trametinib (BrdU incorporation assay).

	Vemurafenib (*µ*M)	Perifosine (*µ*M)	Regorafenib (*µ*M)	Trametinib (nM)
HROC18	8.3	15.4	1.3	39.9
HROC24	0.8	6.7	4.6	5.1
HROC43	9.9	8.7	5.3	89.2
HROC46	12.7	#	2.4	252

#: no inhibitory effects on BrdU incorporation under experimental conditions.
